# Efficacy of digital pupillometry for diagnosis of Horner syndrome

**DOI:** 10.1371/journal.pone.0178361

**Published:** 2017-06-02

**Authors:** Yung Ju Yoo, Hee Kyung Yang, Jeong-Min Hwang

**Affiliations:** 1 Department of Ophthalmology, Kangwon National University Hospital, Kangwon National University Graduate School of Medicine, Chuncheon, Korea; 2 Department of Ophthalmology, Seoul National University College of Medicine, Seoul National University Bundang Hospital, Seongnam, Korea; The University of Melbourne, AUSTRALIA

## Abstract

**Objectives:**

To evaluate the efficacy of digital pupillometry in the diagnosis of anisocoria related to Horner syndrome in adult patients.

**Design:**

Retrospective, observational, case control study.

**Methods:**

Nineteen patients with unilateral Horner syndrome (Horner group) and age-matched controls of 30 healthy individuals with normal vision and neither optic nerve dysfunction nor pupillary abnormalities were included. Pupillary light reflex (PLR) of the Horner group and controls were measured by a dynamic pupillometer (PLR-200; NeurOptics Inc., Irvine, USA). Minimal and maximal (min/max) pupil diameters, latency, constriction ratio, constriction velocity, dilation velocity, and total time taken by the pupil to recover 75% of maximal pupil diameter (T75) were noted. PLR were measured at baseline in both groups and at 30–45 minutes later after 0.5% apraclonidine (Iopidine^®^; Alcon Laboratories, Fort Worth, TX, USA) instillation in the Horner group.

**Main outcome measures:**

The PLR parameters in the affected eye and inter-eye difference before and after 0.5% apraclonidine instillation.

**Results:**

In the Horner group, pupil diameters and T75 showed significant difference between the affected eye and unaffected contralateral eye at baseline (all P<0.00625). Compared to controls, inter-eye difference values of pupil diameters and T75 were significantly larger in the Horner group (all P<0.001). After 0.5% apraclonidine instillation, changes in pupil diameter and constriction ratio were significantly larger in the affected eye compared to the unaffected contralateral eye (all P<0.00625). The area under the receiver operating characteristic curves for diagnosing Horner syndrome were largest for baseline inter-eye difference in min/max pupil sizes (AUC = 0.975, 0.994), T75 (AUC = 0.838), and change in min/max pupil sizes after apraclonidine instillation (AUC = 0.923, 0.929, respectively). The diagnostic criteria for Horner syndrome relying on baseline pupillary measurements was defined as one of the two major findings; 1) smaller maximal pupil diameter in the affected eye with an inter-eye difference of > 0.5 mm, or 2) T75 > 2.61 seconds in the affected eye, which showed a sensitivity of 94.7% and specificity of 93.3%. The diagnostic accuracy of apraclonidine testing showed a sensitivity of 84.6% and specificity of 92.3%.

**Conclusions:**

Digital pupillometry is an objective method for quantifying PLR. Baseline inter-eye difference in maximal pupil sizes and dilation lag measured by T75 was equally effective in the diagnosis of Horner syndrome compared to the reversal of anisocoria after apraclonidine instillation.

## Introduction

Horner syndrome results from injury of the oculosympathetic pathway and is classically described as a clinical triad; ipsilateral ptosis, pupillary miosis, and facial anhydrosis [1, 2]. However, all three symptoms are not always present and the findings are often subtle [2]. Therefore, the diagnosis is confirmed by pharmacologic testing such as cocaine, hydroxyamphetamine, and apraclonidine [2–5]. As the availability of cocaine is limited, apraclonidine (Iopidine^®^; Alcon Laboratories, Fort Worth, TX, USA), a strong α2 and weak α1 adrenergic agonist has been widely used as an alternative [5]. Reversal of anisocoria is found in 30 minutes after the instillation of 0.5% apraclonidine due to upregulation of the α1 receptor in a miotic eye due to a lack of sympathetic input [6, 7].

Although apraclonidine test is a highly sensitive and specific tool for diagnosing Horner syndrome [8, 9], it is still operator dependent because the pupil diameters and pupil light reflex (PLR) are subjectively determined by the examiner. In addition, previous studies reported that apraclonidine testing was positive within 1 week of carotid artery dissection [6] and 1.5 days of central causes such as thalamic hemorrhage [10], there has been no consensus how early apraclonidine will be positive in the setting of postoperative cases or more peripheral lesion. Therefore, a diagnostic tool that does not require pharmacological testing may be beneficial in clinical practice.

There had been few studies reporting the objective quantification of PLR in Horner syndrome [11, 12]. Smith et al.[11] compared the pupil redilation time between Horner’s syndrome patients and healthy subjects using infrared TV pupillometry. However, this device did not provide digitalized parameters. Recently, digital pupillometry has been developed and allows quantification of PLR parameters in objective manner [13–15]. Dilation lag and inter-eye difference of PLR in Horner syndrome could be quantified by digital pupillometry and may help clinicians to distinguish it from other physiologic anisocoria without pharmacologic test. In addition, these tools can make researchers and neuro-ophthalmologists to easily interact with each other.

In the present study, we investigated the efficacy of digital pupillometry for quantifying the PLR at baseline and after apraclonidine instillation to determine the effectiveness of this method as a reliable tool for diagnosing anisocoria related to Horner syndrome.

## Materials and methods

### Study subjects

We retrospectively analyzed patients who were diagnosed with Horner syndrome in the neuro-ophthalmology unit of Seoul National University Bundang Hospital (SNUBH) between January 2011 and June 2016. All patients received a full workup, including complete ophthalmic examination, neurologic imaging tests including contrast-enhanced brain magnetic resonance imaging, carotid doppler ultrasound and neck and thoracic computed tomography angiogram, and apraclonidine tests for Horner syndrome. Ophthalmic examination included visual acuity assessment, automated refraction, slit lamp biomicroscopy, and dilated fundus examination to exclude other pathologic causes that might affect the PLR in both eyes such as glaucoma, vision affected cataracts, mechanical iris dysfunction, and retinopathies. Medication history of drugs affecting PLR, such as pilocarpine, atropine, selective serotonin reuptake inhibitors, and non-selective serotonin reuptake inhibitors were also evaluated. Diagnosis of Horner syndrome was confirmed by two neuro-ophthalmologists (H.K.Y and J.M.H) on the basis of definite clinical history, presence of ptosis, ipsilateral miosis and ipsilateral dilation lag, a positive response after 0.5% apraclonidine test and exclusion of other causes of anisocoria or ptosis [7, 16]. Ptosis was defined as follows: 1) the margin reflex distance is less than 2 mm from the midpupil; or 2) there is 2 mm or more asymmetry between the levels of the upper eyelids, even if both eyelids are 2 mm or more from the midpupil [17].

We selected age-matched controls from individuals with normal vision and no optic nerve dysfunction who had performed the digital pupillometry at the outpatient clinic of SNUBH. Subjects diagnosed as physiologic anisocoria which the inter-eye difference of pupil diameter is greater than 0.5mm were excluded. To verify that there is no significant inter-eye difference in PLR parameters of the normal population measured with digital pupillometry, we investigated PLR of 30 healthy controls. We also used these results as standard values for detection of abnormal PLR in the affected eye of the Horner group. The individual in this manuscript has given written informed consent (as outlined in PLOS consent form) to publish the case details. The study was approved by the Institutional Review Board of Seoul national university Bundang hospital and adheres to the tenets of the Declaration of Helsinki.

### Pupillary light reflex measurements by digital infrared pupillometry

PLR were obtained and recorded with the PLR-200 Pupillometer (NeurOptics Inc., Irvine, USA). PLR-200 pupillometer is an automated monocular infrared pupillometer that records pupil images of each eye separately. PLR of each subject were measured in a consistent order of right eye followed by the left eye. Pupillometry was performed after 3 minutes of dark adaption. Patients were instructed to fixate on a small target object such as a dim flash light at least 3 meters away with the contralateral eye. PLR-200 pupillometer has an eyecup designed for fitting periorbital area which helps reduce the possibility of light entering the tested eye and standardize stimulus distance and intensity [18]. Stimuli consisted of pulses of light with as fixed intensity of 180 microwatts/cm^2^ and duration of 185 milliseconds. Pupil size measurements were sampled at a frequency of 32 frames per second and lasted up to 5 seconds, allowing a full or partial recovery of the pupil size after light constriction. PLR of each eye was measured twice and the average of data was used. The device has been specifically designed to minimize possible inter-observer variability in the pupillary evaluation.

### Parameters of pupillary light reflex

**Fig 1:**
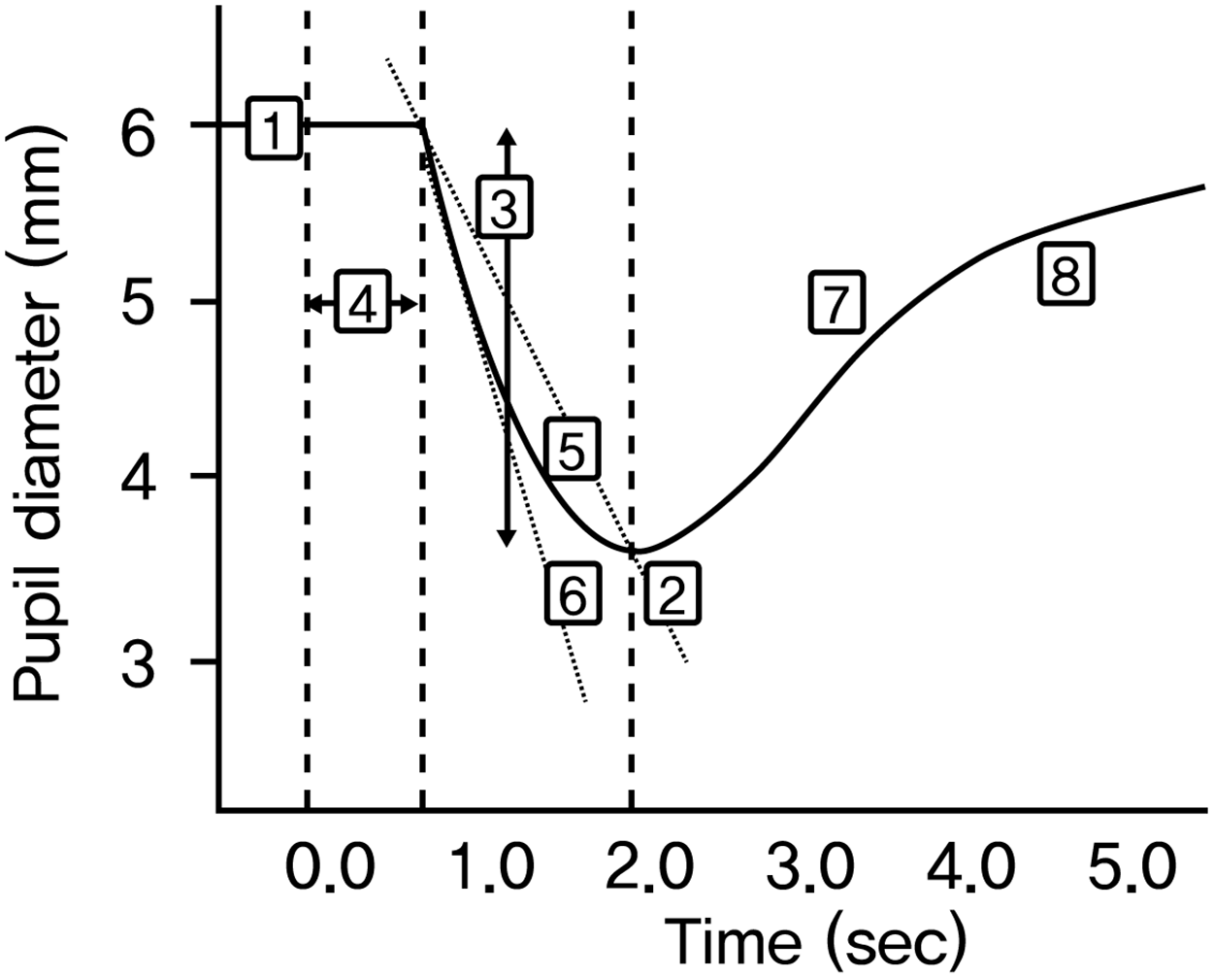
Schematic diagram of the pupillary light response illustrating the recorded pupillary light reflex parameters. 1) maximal pupil diameter, 2) minimal pupil diameter, 3) pupil constriction ratio, 4) constriction latency, 5) average constriction velocity, 6) maximal constriction velocity, 7) average dilation velocity, 8) total time taken by the pupil to recover 75% of maximal pupil diameter.

Eight PLR parameters were presented with pupil response curves [18]. The maximal pupil diameter (mm) was defined as the initial resting pupil size and minimal pupil diameter (mm) as the smallest pupil size during constriction. The pupillary constriction ratio (%) was defined as the difference between the maximum and minimum diameters divided by the maximal pupil diameter, and the latency (sec) as the time difference between initiation of retinal light stimulation and onset of pupillary constriction. Average constriction velocity (ACV, mm/sec) was defined as the amplitude of pupil constriction divided by the duration of constriction and average dilation velocity (ADV, mm/sec) as the amount of pupil size dilation after constriction divided by the duration of recovery to maximal pupil diameter. Maximal constriction velocity (MCV) was defined as the peak value of the velocity during constriction which is larger than the ACV. Total time from the peak of the constriction to the recovery of the pupil to recover 75% of maximal pupil diameter (T75) was also measured. Fig 1 is a schematic diagram of the pupillary reaction curve illustrating the recorded PLR parameters.

### Topical apraclonidine test

The apraclonidine test was done as described by Koc et al [9]. First, baseline pupil diameter was recorded in normal room illumination and a dark room. The pupil diameter recorded in a dark room corresponds to the data presented in the results section. Baseline PLR was measured with digital pupillometry in each eye before applying 0.5% apraclonidine eyedrops (Iopidine^®^; Alcon Laboratories, Fort Worth, TX, USA). At 30–45 minutes after one drop of 0.5% apraclonidine was applied in both eyes, post-instillation PLR measurements were repeated. The positive results were defined as a change of more than 0.5 mm in maximal pupil diameters compared to baseline after apraclonidine administration.

### Statistical analysis

The comparison of PLR parameters and ocular characteristics between patients and age-matched controls were performed using the Mann-Whitney U test. Paired *t*-test was conducted to determine whether there was a statistically significant inter-eye difference between the PLR of patients both at baseline and after apraclonidine instillation. For each PLR parameter, the absolute value of inter-eye difference was compared with the absolute measurements of the control group. The usefulness of PLR parameters in diagnosing Horner syndrome was assessed using the area under the receiver operating characteristic curve (AUC). Analyses were performed using the Statistical Package for the Social Sciences (version 21.0; SPSS, Chicago, IL, USA) and R-statistics (v2.15.1 software for Macintosh; R Foundation for Statistical Computing, Vienna, Austria). The statistical analyses are performed according to the paired t-test with Bonferroni adjustment. According to Bonferroni-adjustment, results are considered statistically significant when two-sided P-values are less than 0.00625 (0.05/8 Bonferroni adjustment). Data are presented as mean ± standard deviation.

## Results

Nineteen patients with unilateral Horner syndrome (Horner group) were included in this study. All 19 patients were Korean and their average age was 43.0 ± 14.1 years (range 17.9–72.9 years). Among them, 8 patients (42.1%) were diagnosed with iatrogenic Horner syndrome, 1 patient due to carotid dissection, and 1 patient related to cavernous sinus hemangioma. Sixteen patients (84.2%) had ipsilateral ptosis and the mean upper eyelid margin reflex distance of the affected eye and contralateral normal eye was 1.7 and 3.6 mm, respectively (P < 0.001, paired t-test). Among 16 patients with reliable history taking and physical examination, 37.5% (6/16) of patients reported ipsilateral anhydrosis.

Thirty healthy control subjects (mean age 43.0 ± 14.1 years) (control group) were also included for comparison. The mean age of the Horner group and control group were similar (P = 0.975, unpaired t-test). Female to male ratios were 1.38 (11/8) for the Horner group and 0.67 (12/18) for the control group (P = 0.254, Chi square test). All PLR parameters of controls showed no inter-eye differences (all P > 0.15, paired t-test). Comparison of PLR parameters at baseline between controls and the contralateral unaffected eye of the Horner group revealed no significant difference (all P > 0.07, Mann-Whitney U test).

### Inter-eye difference of pupil response parameters for patients with Horner syndrome

Table 1 compares the inter-eye differences of baseline PLR parameters. Relative to contralateral unaffected eyes, maximal and minimal pupil diameters were smaller in affected eyes (both P < 0.001). In the constriction phase, constriction ratio, constriction latency, ACV, and MCV did not show significant inter-eye differences after Bonferroni correction (P = 0.013, 0.053, 0.112 and 0.282, respectively, paired t-test). Conversely, in the dilation phase, T75 of the affected eyes were significantly longer compared with the unaffected eyes after Bonferroni correction (P = < 0.001, paired t-test).

**Table 1:** Comparison of pupil response in Horner syndrome patients between affected eyes and non-affected contralateral eyes.

	Baseline			Post-apraclonidine test		
	Horner eye	Contralateral eye	P value*	Horner eye	Contralateral eye	P value*
Maximal pupil diameter (mm)	4.5 ± 0.9 (2.9,6.3)	5.6 ± 0.8 (3.8,7.1)	**<0.001**	5.5 ± 1.0 (3.7,7.0)	5.0 ± 0.8 (3.4,6.1)	0.033
Minimal pupil diameter (mm)	3.0 ± 0.7 (1.8,4.3)	3.9 ± 0.7 (2.3,4.8)	**<0.001**	3.9 ± 0.9 (2.4,5.2)	3.3 ± 0.6 (2.4,4.0)	0.014
CON (%)	33.0 ± 3.4 (28,40)	31.1 ± 4.0 (24,39)	0.013	27.8 ± 5.6 (17,35)	33.3 ± 5.0 (24,39)	0.007
Latency (sec)	0.23 ± 0.02 (0.19,0.28)	0.24 ± 0.02 (0.22,0.28)	0.053	0.25 ± 0.04 (0.19,0.31)	0.24 ± 0.02 (0.22,0.28)	0.219
ACV (mm/s)	3.30 ± 0.50 (2.32,4.01)	3.50 ± 0.50 (2.69,4.68)	0.112	2.90 ± 0.55 (2.20,3.82)	3.51 ± 0.62 (2.65,4.25)	**<0.001**
MCV (mm/s)	4.35 ± 0.76 (3.12,5.65)	4.51 ± 0.73 (3.31,5.78)	0.282	3.86 ± 0.84 (2.63,5.50)	4.54 ± 0.84 (3.43,5.91)	**0.005**
ADV (mm/s)	0.83 ± 0.14 (0.54,1.01)	0.95 ± 0.19 (0.60,1.41)	0.027	0.74 ± 0.13 (0.48,0.95)	0.92 ± 0.14 (0.68,1.09)	**<0.001**
T75% (sec)	3.09 ± 1.02 (1.54,4.17)	1.84 ± 0.77 (0.68,3.70)	**<0.001**	2.16 ± 0.95 (0.86,4.00)	2.99 ± 1.77 (0.68,2.99)	0.064

ACV = Average constriction velocity; ADV = Average dilation velocity; CON = Pupil constriction ratio; MCV = Mean constriction velocity; T75 = Total time from the peak of the constriction to the recovery of the pupil to 75% of maximal pupil diameter.

* P value by paired t test.

Data are presented as mean ± standard deviation (range). Factors with statistical significance are shown in boldface. A significance level of P = 0.00625 (0.05/8 Bonferroni adjustment) was used to adjudge whether any PLR parameters were significantly different between two groups.

Table 1 also compares PLR parameters after apraclonidine test between affected eyes and contralateral unaffected eyes. After apraclonidine instillation, the affected pupils showed an increase in maximal and minimal pupil diameter instead of the decrease shown in contralateral unaffected eyes (P = 0.033 and 0.014, respectively, paired t-test).

Table 2 compares inter-eye difference values of baseline PLR parameters between the Horner group and control group. Difference of maximal pupil diameter, minimal pupil diameter, and T75 were significantly larger in the Horner group compared to the control group (all P < 0.001, Mann-Whitney U test).

**Table 2:** Inter-eye difference of baseline pupil response between Horner syndrome and controls.

	Inter-eye difference in Horner syndrome	Absolute inter-eye difference in controls	P value*
Maximal pupil diameter (mm)†	1.1 ± 0.6 (0.2,2.3)	0.2 ± 0.1 (0.0,0.5)	**<0.001**
Minimal pupil diameter (mm)^†^	0.9 ± 0.4 (0.4,1.8)	0.1 ± 0.1 (0.0,0.5)	**<0.001**
CON (%)‡	2.2 ± 3.2 (-6,6)	1.4 ± 1.0 (0,3.0)	0.364
Latency (sec)^†^	0.01 ± 0.03 (0.0,0.03)	0.02 ± 0.02 (0.0,0.06)	0.366
ACV (mm/s)^†^	0.19 ± 0.48 (-0.41,1.30)	0.23 ± 0.14 (0.03,1.05)	0.246
MCV (mm/s)^†^	0.16 ± 0.60 (-0.90,1.43)	0.29 ± 0.26 (0.01,1.27)	0.352
ADV (mm/s)^†^	0.12 ± 0.22 (-0.16,0.71)	0.15 ± 0.13 (0.01,0.60)	0.529
T75% (sec)‡	1.18 ± 0.95 (-0.31,2.66)	0.25 ± 0.14 (0.03,0.52)	**<0.001**

ACV = Average constriction velocity; ADV = Average dilation velocity; CON = Pupil constriction ratio; MCV = Mean constriction velocity; T75 = Total time from the peak of the constriction to the recovery of the pupil to 75% of maximal pupil diameter. Data are presented as mean ± standard deviation (range). Factors with statistical significance are shown in boldface. A significance level of P = 0.00625 (0.05/8 Bonferroni adjustment) was used to adjudge whether any PLR parameters were significantly different between two groups.

* P value by Mann-Whitney U test

^†^ The difference was calculated as healthy eye minus affected eye

^‡^ The difference was calculated as affected eye minus healthy eye

### Diagnostic performance of each pupil response parameter for Horner syndrome

Table 3 compares changes in PLR parameters measured with digital pupillometry after apraclonidine instillation between the affected eye and the contralateral normal eye in the Horner group. Changes in maximal pupil diameters between baseline and post apraclonidine tests were significantly lager in the affected eye (1.1 ± 0.8 mm), compared to the unaffected eye (-0.4 ± 0.4 mm) (P < 0.001, paired t-test). Pupil constriction ratio, ACV, and MCV decreased after apraclonidine test in the affected eye compared to the unaffected eye which revealed no significant difference after Bonferroni correction (P = 0.014, 0.011 and 0.035, paired t-test). A positive apraclonidine test measured with digital pupillometry was noted in 84.6% in the affected eye and in 7.6% in contralateral normal eye (P < 0.001).

**Table 3:** Changes in pupil response parameters measured by digital pupillometry after 0.5% apraclonidine instillation in patients with Horner syndrome.

	Affected eye	Contralateral eye	P value*
Maximal pupil diameter (mm)	1.1 ± 0.8 (-0.4,2.3)	-0.4 ± 0.4 (-1.0,0.1)	**<0.001**
Minimal pupil diameter (mm)	1.0 ± 0.8 (-1.8,0.4)	-0.4 ± 0.4 (-1.1,0.1)	**<0.001**
CON (%)	-5.8 ± 5.0 (-14,2)	2.3 ± 4.7 (-4,13)	**0.001**
Latency (sec)	0.02 ± 0.03 (-0.03,0.06)	-0.01 ± 0.01 (-0.03.0.0)	0.014
ACV (mm/s)	-0.47 ± 0.66 (-1.36,0.88)	0.05 ± 0.37 (-0.44,0.56)	0.011
MCV (mm/s)	-0.58 ± 0.91 (-1.89,1.11)	0.05 ± 0.56 (-0.93,0.85)	0.035
ADV (mm/s)	-0.12 ± 0.20 (-0.12,0.46)	-0.07 ± 0.15 (-0.38,0.14)	0.397
T75% (sec)	-0.64 ± 1.14 (-2.4,0.5)	0.11 ± 0.81 (-1.63,1.90)	0.062

ACV = Average constriction velocity; ADV = Average dilation velocity; CON = Pupil constriction ratio; MCV = Mean constriction velocity; T75 = Total time from the peak of the constriction to the recovery of the pupil to 75% of maximal pupil diameter. Data are presented as mean ± standard deviation (range). Factors with statistical significance are shown in boldface. A significance level of P = 0.00625 (0.05/8 Bonferroni adjustment) was used to adjudge whether any PLR parameters were significantly different between two groups.

* P value by paired t-test

**Fig 2:**
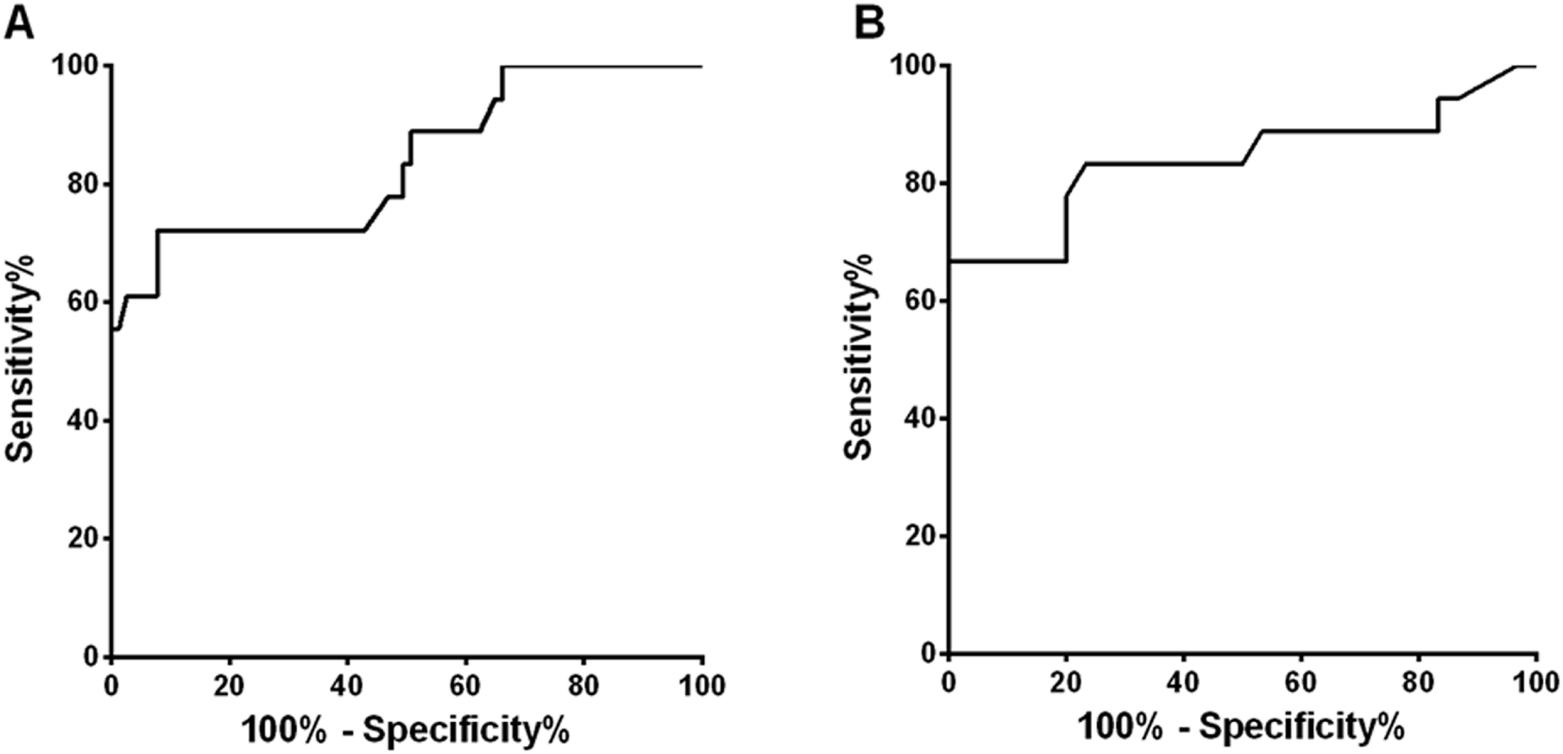
Receiver operating characteristic curve of the baseline total time from the peak of constriction to the recovery of 75% of maximal pupil diameter (T75) and baseline inter-eye difference of T75. The maximum area under the curve were 0.838 (95% Confidence interval (CI), 0.720 to 0.956; P < 0.0001) and 0.840 (95% CI, 0.702–0.978; P < 0.0001). With a cutoff value of T75> 2.61 sec, the sensitivity and specificity of the baseline T75 was 72.2% and 92.2%, respectively. As for the baseline inter-eye difference of T75, the sensitivity and specificity were 77.8% and 80.0% with a cutoff value of > 0.31 sec.

The performance of each parameter for diagnosing Horner syndrome was assessed using the AUC (Table 4). The best baseline parameters for diagnosing Horner syndrome other than inter-eye differences in pupil diameters (Maximal and minimal pupil diameter) were baseline T75 (AUC = 0.838) and baseline inter-eye difference of T75 (AUC = 0.840) (Fig 2). With a cutoff value of 2.61 sec, the sensitivity and specificity of the baseline T75 was 72.2% and 92.2%, respectively. As for the baseline inter-eye difference of T75, the sensitivity and specificity were 77.8% and 80.0% with a cutoff value of 0.31 sec. If PLR parameters meet both criteria of T75 (baseline T75 > 2.61 sec and inter-eye difference of T75 > 0.31 sec) the sensitivity and specificity were 68.4% and 96.7%, respectively.

**Table 4:** AUC of pupil response parameters to discriminate between Horner syndrome and control.

	AUC	95% CI	Cut off value	Sensitivity (%)	Specificity (%)
**Baseline**					
Maximal pupil diameter (mm)	0.898	0.687–0.908	5.2	84.2	84.2
Minimal pupil diameter (mm)	0.802	0.690–0.905	3.4	68.4	83.3
CON (%)	0.630	0.488–0.771			
Latency (sec)	0.513	0.361–0.665			
ACV (mm/s)	0.694	0.564–0.824			
MCV (mm/s)	0.663	0.517–0.808			
ADV (mm/s)	0.688	0.567–0.810			
T75% (sec)	0.838	0.720–0.956	2.6	72.2	92.2
**Baseline Inter-eye difference**					
Maximal pupil diameter (mm)	0.975	0.936–1.000	0.45	89.5	93.1
Minimal pupil diameter (mm)	0.994	0.979–1.000	0.35	100	96.6
CON (%)	0.765	0.599–0.930			
Latency (sec)	0.572	0.498–0.747			
ACV (mm/s)	0.600	0.388–0.811			
MCV (mm/s)	0.559	0.358–0.760			
ADV (mm/s)	0.517	0.312–0.722			
T75% (sec)	0.840	0.702–0.978	0.31	77.8	80.0
**Post Inter-eye difference**					
Maximal pupil diameter (mm)	0.649	0.394–0.903			
Minimal pupil diameter (mm)	0.701	0.462–0.939			
CON (%)	0.692	0.441–0.943			
Latency (sec)	0.583	0.384–0.783			
ACV (mm/s)	0.536	0.268–0.803			
MCV (mm/s)	0.518	0.267–0.768			
ADV (mm/s)	0.690	0.468–0.913			
T75% (sec)	0.618	0.462–0.777			
**Difference between baseline and post apraclonidine test**					
Maximal pupil diameter (mm)	0.923	0.813–1.000	0.5	84.6	100
Minimal pupil diameter (mm)	0.929	0.825–1.000	0.5	84.6	100
CON (%)	0.608	0.436–0.779			
Latency (sec)	0.775	0.579–0.971			
ACV (mm/s)	0.747	0.542–0.952			
MCV (mm/s)	0.719	0.506–0.931			
ADV (mm/s)	0.615	0.383–0.846			
T75% (sec)	0.636	0.465–0.806			

ACV = Average constriction velocity; ADV = Average dilation velocity; CON = pupil constriction ratio (%); MCV = Mean constriction velocity; T75 = Total time from the peak of the constriction to the recovery of the pupil to 75% of maximal pupil diameter. Sensitivity and specificity values are noted for factors with AUC>0.8.

The diagnostic criteria for Horner syndrome relying on baseline pupillary measurements was defined as one of the two major findings; 1) small maximal pupil diameter with inter-eye difference of > 0.5 mm, or 2) T75 > 2.61 seconds in the affected eye. The sensitivity and specificity of this criteria were 94.7% and 93.3%, respectively for diagnosing Horner syndrome.

Among parameters after apraclonidine instillation, the amount of change in maximal and minimal diameters reflecting the ‘reversal of anisocoria’, and pupil constriction ratio after administration of apraclonidine showed the highest AUCs (AUC = 0.923, 0.929, and 0.910 respectively). The diagnostic accuracy of apraclonidine testing for diagnosing Horner syndrome showed a sensitivity of 84.6% and specificity of 92.3%.

### Representative case

**Fig 3:**
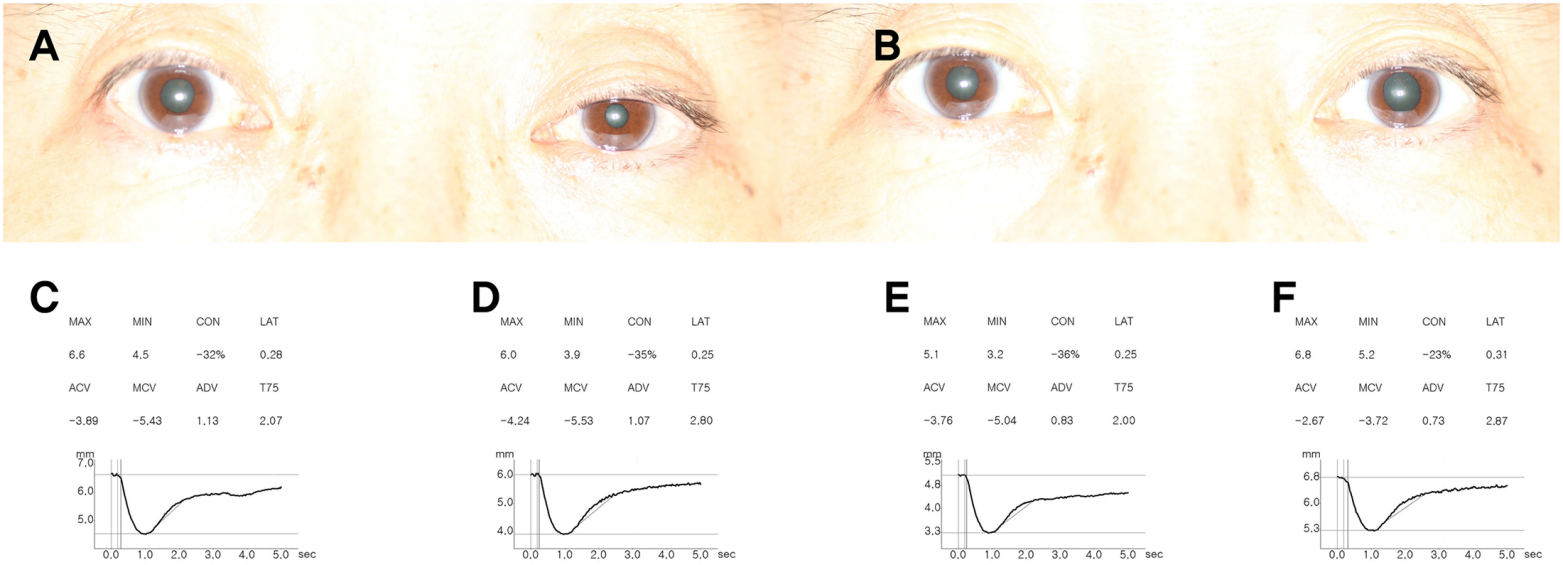
A patient diagnosed with iatrogenic Horner syndrome in the left eye after total thyroidectomy (A-F). A, The patient at baseline, showing left ptosis and miosis; B, Thirty-five minutes after 1 drop of 0.5% apraclonidine instillation in both eyes. Note reversal of baseline anisocoria. C-F, Eight PLR parameters were presented with pupil response curves. The pupillary constriction ratio (CON) was defined as the minimal pupil diameter divided by the maximal pupil diameter, and the latency (LAT) as the time difference between initiation of retinal light stimulation and onset of pupillary constriction. Average constriction velocity (ACV), maximal constriction velocity (MCV), average dilation velocity (ADV) and total time taken by the pupil to recover 75% of maximal pupil diameter (T75) was also presented. C and D, Baseline pupil light reflex (PLR) curve measured with digital pupillometry in both affected eye (D) and contralateral normal eye (C). Note that inter-eye difference in maximal pupil diameter (6.0 mm in affected left eye and 6.6 mm in unaffected right eye), ADV and T75; ADV of the affected eyes (1.07 mm/sec) was slower than that of the unaffected eyes (1.13 mm/sec) and T75 of the affected eyes (2.87 sec) was longer compared with the unaffected eyes (2.07 sec). E and F, PLR curve after 0.5% apraclonidine instillation showed definite change of pupil diameter in affected eye compared to contralateral normal eye; the affected pupils showed an increase in maximal and minimal pupil diameter (6.8 mm and 5.2 mm, respectively) instead of decrease shown in contralateral unaffected eyes (5.1 mm and 3.2 mm, respectively). ADV of the affected eyes (0.73 mm/sec) was decreased and T75 of the affected eyes (3.10 sec) was increased after apraclonidine instillation.

Fig 3 shows a representative case of a patient diagnosed with iatrogenic Horner syndrome in the left eye. Miosis, anisocoria and pupil enlargement after apraclonidine test are objectively quantified by digital pupillometer measurements and reversal of baseline anisocoria is evident by the measurements of digital pupillometry after apraclonidine test.

## Discussion

This study demonstrated the diagnostic efficacy of quantitative analysis of the PLR using digital pupillometry in unilateral Horner syndrome patients. Using digital pupillometry, patients with Horner syndrome demonstrated distinct inter-eye difference in PLR parameters compared to normal controls. At baseline, pupil diameters and the time for redilation (T75) are significantly different between both eyes in the Horner group. After apraclonidine instillation, in addition to the reversal of anisocoria, constriction velocity decreased in the affected eye unlike those of the contralateral eye which remained unchanged. Baseline inter-eye difference in maximal pupil sizes and dilation lag measured by T75 was equally effective in the diagnosis of Horner syndrome compared to the reversal of anisocoria after apraclonidine instillation.

Using digital pupillometry, the AUCs for diagnosing Horner syndrome were largest for baseline inter-eye difference in maximal and minimal pupil sizes (AUC = 0.975, 0.994), T75 (AUC = 0.838), baseline inter-eye difference of T75 (AUC = 0.840), and change in maximal and minimal pupil sizes after apraclonidine instillation (AUC = 0.923, 0.929, respectively). The AUC of baseline inter-eye differences in pupil diameter was greater than 0.9, indicating that the sensitivity of the digital pupilometer is reliable. Baseline data show good sensitivity and specificity, however, they cannot be used to confirm the diagnosis of Horner syndrome. The AUC of baseline T75 was larger than 0.8 without apraclonidine testing. T75 is a baseline PLR parameter which reflects dilation lag, and this can be used as the diagnostic criteria without pharmacologic testing. The diagnostic sensitivity was highest when one of the two major findings were satisfied; 1) smaller maximal pupil size in the affected eye with an inter-eye difference of > 0.5 mm, or 2) T75 > 2.61 sec in the affected eye. The sensitivity of this criterion is similar to the previously reported apraclonidine test which ranged from 88% to 96.5% [8, 16]. In the present study, there was only one patient with a false negative result according to the above criteria (inter-eye difference 0.4mm, T75 was 1.79sec). This patient developed Horner syndrome due to cavernous sinus hemangioma and a false negative result may be because pupillometry was performed only 2 days after symptom onset. Moreover, this patient also showed a negative result in the apraclonidine test. False-negative results of the apraclonidine test may be found in acute cases of Horner syndrome because up regulation of α1-receptors takes between 5 and 8 days to develop [19]. A future study may be required to establish the change in baseline measurements of pupil sizes and T75 by digital pupillometry according to the time after onset of Horner syndrome.

Pupil with damage of the oculosympathetic pathway shows slow and delayed dilation in darkness, which has been called “dilation lag” [20]. Previously, there have been various attempts to objectively quantify the dilation lag using a camcorder or a computerized binocular pupillometer [14, 20]. Sylvain et al.[20] defined dilation lag as more than 0.4 mm asymmetry of inter-eye difference of pupil diameter between five seconds in darkness and 15 seconds in darkness. However, assessment of dilation lag using this definition revealed a low sensitivity (53%). There is a possibility of underestimating the constriction in patients with small pupils [15] which could induce false negative results. In the present study, results of digital pupillometry showed distinct inter-eye difference in two parameters (ADV, T75) in the dilation phase which can be explained by the dilation lag. Digital pupillometry can objectively measure dilation velocity (ADV) as well as the time for the pupil to recover 75% of the maximal diameter in the dilatation phase (T75). As T75 relies on the relative ratio of pupil measurements instead of the absolute pupil size, the inter-individual pupil size variation can be ignored and this shortcoming of previous methods can be overcome by using digital pupillometry.

Our study also supports the validity of apraclonidine testing for diagnosing Horner syndrome. The mydriatic effect which is observed in the affected eye of Horner syndrome is explained by upregulation of the α1 adrenergic receptors on iris dilator muscles in the absence of the normal sympathetic tone [1, 18, 21]. Almost all previous studies demonstrated the efficacy of apraclonidine testing based on reversal of anisocoria which was calculated photographically [7, 8, 16]. In the present study, we objectively compared the constriction ratio, constriction latency, and average and maximal constriction velocities of the affected eye with those of the contralateral normal eye using digital pupillometry.

There are some limitations in the present study. First, there are no normative data provided of the PLR parameters obtained from digital pupillometry. However, we overcame this problem by including healthy control subjects. Second, as most of the patients were identified from a single institution, there may be some selection bias in the etiology of Horner syndrome. Third, this study included patients with acquired Horner syndrome of which half of the patients had a history of surgery. Therefore, our study results may not be applicable to congenital Horner syndrome or acquired Horner syndrome caused by other reasons except iatrogenic injury of the sympathetic pathway.

In conclusion, our results show that PLR measured with digital pupillometry revealed distinct inter-eye difference in Horner syndrome both at baseline and after apraclonidine 0.5% test. Baseline inter-eye difference in maximal pupil sizes and dilation lag measured by T75 was equally effective in the diagnosis of Horner syndrome compared to the reversal of anisocoria after apraclonidine instillation. Evaluation of PLR using digital pupillometry is a simple, fast, specific, and reliable test and provides objective and quantitative information for the diagnosis of Horner syndrome.
